# Clinical predictors and noninvasive imaging in Fontan-associated liver disease: A systematic review and meta-analysis

**DOI:** 10.1097/HC9.0000000000000580

**Published:** 2024-11-15

**Authors:** Jae Hee Seol, Jinyoung Song, Soo Jin Kim, Hoon Ko, Jae Yoon Na, Min Jung Cho, Hee Joung Choi, Jue Seong Lee, Kyung Jin Oh, Jo Won Jung, Se Yong Jung

**Affiliations:** 1 Department of Pediatrics, Yonsei University Wonju College of Medicine, Wonju, Republic of Korea; 2 Department of Pediatrics, Yonsei University College of Medicine, Seoul, Republic of Korea; 3 Department of Pediatrics, Samsung Medical Center, Sungkyunkwan University School of Medicine, Seoul, Republic of Korea; 4 Department of Pediatrics, Sejong General Hospital, Buchun, Republic of Korea; 5 Department of Pediatrics, Pusan National University Yangsan Hospital, Pusan National University School of Medicine, Yangsan, Republic of Korea; 6 Department of Pediatrics, Hanyang University College of Medicine, Seoul, Republic of Korea; 7 Department of Pediatrics, College of Medicine, Gyeoungsang National University Changwon Hospital, Changwon, Geongsangnam-do, Republic of Korea; 8 Department of Pediatrics, Keimyung University Dongsan Hospital, Keimyung University School of Medicine, Daegu, Republic of Korea; 9 Department of Pediatrics, Korea University Anam Hospital, Korea University College of Medicine, Seoul, Republic of Korea; 10 Department of Pediatrics, Seoul Metropolitan Government-Seoul National University Boramae Medical Center, Seoul, Republic of Korea; 11 Division of Pediatric Cardiology, Department of Pediatrics, Congenital Heart Disease Center, Severance Cardiovascular Hospital, Yonsei University College of Medicine, Seoul, Republic of Korea

**Keywords:** Fontan circulation, hemodynamic changes, liver dysfunction, noninvasive diagnostic method

## Abstract

**Background:**

Despite the development of several imaging modalities for diagnosing Fontan-associated liver disease (FALD), there is no optimal protocol for the follow-up of FALD. We conducted a systematic review and meta-analysis to identify factors related to liver fibrosis using biopsy reports and to identify alternative noninvasive modalities that could better reflect liver histological changes in FALD.

**Methods:**

A systematic review and meta-analysis were conducted following the PRISMA guidelines Table S2. We searched Embase, PubMed, and Cochrane databases for studies on FALD, focusing on those assessing clinical factors associated with liver fibrosis severity through liver biopsy and noninvasive imaging techniques.

**Results:**

A total of 42 studies were identified, of which 12 conducted meta-analyses and subgroup analyses of the severity of liver fibrosis using liver biopsies. Liver biopsy results showed a weak positive correlation between Fontan duration and fibrosis severity (*R* = 0.36). Subgroup analyses revealed significant differences in hemodynamic parameters, such as Fontan pressure, between patients with mild and severe fibrosis. Platelet count, aspartate aminotransferase to platelet ratio index, and fibrosis-4 index were significantly associated with fibrosis severity, with severe fibrosis showing lower platelet counts and higher aspartate aminotransferase to platelet ratio index and fibrosis-4 index levels. Noninvasive imaging modalities, particularly magnetic resonance elastography and shear wave elastography, demonstrated strong correlations with biopsy-confirmed fibrosis severity.

**Conclusions:**

This study identifies key clinical factors, and noninvasive modalities accurately reflect liver fibrosis severity in patients with FALD. Clinical factors such as platelet count, aspartate aminotransferase to platelet ratio index, and fibrosis-4 index may aid in identifying patients at risk for severe fibrosis. In addition, magnetic resonance elastography and shear wave elastography are promising tools for noninvasive assessment in our study. Further research is needed to refine these diagnostic approaches and improve patient management.

## INTRODUCTION

A functional single ventricle occurs when only 1 ventricle develops, resulting in the mixing of all blood within the ventricle before circulation. This physiology arises from tricuspid atresia, mitral atresia, hypoplastic right or left ventricular syndrome, or the impossibility of biventricular repair due to other cardiac defects. The Fontan operation, first described in 1971 for tricuspid atresia, separates the pulmonary and systemic circulation by creating a venous-to-pulmonary connection.^1^ This allows passive delivery of deoxygenated blood to the lungs and uses the single ventricle for systemic circulation, serving as a palliative strategy for single-ventricle physiology. As of 2020, the estimated number of patients with Fontan circulation is ~50,000, which is equivalent to 66 per million. This number is expected to increase gradually in the future.^2^

In Fontan circulation, the nonpulsatile and passive flow of venous blood into the pulmonary arteries leads to elevated central venous pressure (CVP) and reduced cardiac output.^3^,^4^,^5^ Persistent CVP elevation causes congestive hepatopathy and histological changes, marked by elevated bilirubin and GGT. These hemodynamic changes persist as long-term sequelae, and continued exposure to low cardiac output states results in hepatic ischemia. This ischemia promotes the release of profibrotic factors, leading to sinusoidal fibrosis and the formation of regenerative nodules, often resulting in HCC. As liver function continues to deteriorate, albumin levels decrease, and both coagulopathy and thrombocytopenia manifest.^5^,^6^ These histological changes and dysfunctions caused by hemodynamic changes and systemic venous congestion after Fontan surgery are known as Fontan-associated liver disease (FALD).

No established medical therapies currently exist for FALD, but early diagnosis allows interventions to optimize Fontan circulation, potentially preventing or slowing the progression to advanced cirrhosis. Liver biopsy, laboratory tests, and various screening modalities, including imaging studies and elastography, were employed to assess FALD progression. Noninvasive imaging methods, such as ultrasound, CT, and MRI, may be viable options for screening and detecting chronic liver disease and HCC.^7^ Furthermore, elastography is a novel noninvasive method for assessing liver fibrosis based on liver stiffness.

The optimal protocol for early diagnosis and follow-up of FALD remains challenging. Although liver biopsy is the gold standard for diagnosing FALD, its use as a screening tool is limited by bleeding risks, infections, and sampling errors. Therefore, there is a pressing need to identify reliable noninvasive diagnostic methods that can accurately assess FALD progression and potentially replace liver biopsy.

Given the challenges in diagnosing FALD and the limitations of current methods, this study aimed to review the existing literature and studies to identify factors contributing to FALD severity and to determine the most effective noninvasive diagnostic methods for patients with prolonged Fontan circulation.

## METHODS

A systematic review and meta-analysis were conducted according to the current Preferred Reporting Items for Systematic Reviews and Meta-Analyses (PRISMA) guidelines.^8^ The protocol for this study was registered in the International Prospective Register of Systematic Reviews (PROSPERO) before data extraction (CRD42024535578).

### Search strategy

Inclusion criteria included studies involving patients of all ages who underwent Fontan surgery, with liver disease diagnosed by liver biopsy, and/or performed imaging modalities. We did not impose any restrictions based on geographic location, ethnicity, study design, year, or language of publication. We searched online databases for articles on FALD using Embase, PubMed, and Cochrane Central Register of Controlled Trials. The search, conducted on February 20, 2024, used Medical Subject Headings terms like “Fontan procedure,” “univentricular heart/surgery,” “ultrasonography,” “elasticity imaging techniques,” “magnetic resonance imaging,” “tomography,” and “X-ray computed”. The details of the full search strategy are in the Supplemental Material, Table S1, http://links.lww.com/HC9/B74.

### Exclusion criteria

Articles were excluded if one or more of the following criteria were met: (1) case reports, review articles, editorials, correspondence, notes, comments, and letters; (2) a study targeting patient groups with high selection bias; and (3) a study including a group of patients who received liver transplantation.

### Data collection process

All articles were independently screened for eligibility by 2 of the 3 authors (Seol Jae Hee and Jung Se Yong). Initially, the titles and abstracts were screened, followed by a full-text review of the selected articles. Data were extracted using a predesigned standardized form. In cases of disagreement, a consensus was reached between the 2 reviewers (Seol Jae Hee and Jung Se Yong), and any remaining discrepancies were resolved through discussion with a third reviewer (Jung Jo Won).

We extracted the following data: (1) title, (2) authors, (3) year of publication, (4) type of study, (5) number of patients included in the study, (6) patient population (adults, pediatrics, or both), (7) age at follow-up/biopsy/diagnosis, (8) age at Fontan surgery, (9) time since the Fontan operation, (10) interventions, (11) reason for liver biopsy if reported, (12) histological scoring system, and (13) clinical data (hemodynamic and laboratory data).

### Study objectives

The primary outcome was the identification of clinical factors associated with the severity of FALD diagnosed by liver biopsy. In these studies, the demographic, hemodynamic, and biochemical factors related to Fontan severity were analyzed. An additional objective was to discover noninvasive modalities that strongly correlate with biopsy findings. We classified the studies by diagnostic modality and described the outcomes of the relationship between hemodynamic and biochemical factors.

### Synthesis methods and statistical analysis

The meta-analysis was performed using R software (version 4.3.13) and the Meta package (version 7.0-0) (R Core Team). Subgroup analysis was performed on the outcomes in the mild and severe liver fibrosis groups. If substantial heterogeneity was observed due to patient characteristics, analyses were conducted separately for pediatric and adult populations. Statistical analyses, including the random-effects model, were conducted to account for both within-study and between-study variance. In addition, weighted averages were calculated to give more importance to studies with larger sample sizes or higher quality. Pooled estimates with 95% CIs were calculated using a random-effects model and visualized using a forest plot. Statistical heterogeneity was evaluated using the *I*^2^ statistics.^9^ An *I*^2^ value below 25% indicated low heterogeneity, while over 75% was considered high. Significance was set at a *p* value <0.05.

### Quality assessment

The risk of bias assessment used the tool for nonrandomized studies (RoBANS) proposed by Seo et al^10^ and included the assessment of 8 main domains: participant comparability, selection of participants, confounding variables, measurement of exposure, blinding of outcome assessments, outcome evaluation, incomplete outcome data, and selective reporting. The RoBANS is a validated, reliable, and feasible tool for assessing the methodological quality of nonrandomized studies. Review Manager version 5.3.3 (RevMan for Macos, Nordic Cochrane Centre) generated a summary of the RoBANS results. Supplemental Table S2, http://links.lww.com/HC9/B74.

## RESULTS

### Search results

**FIGURE 1:**
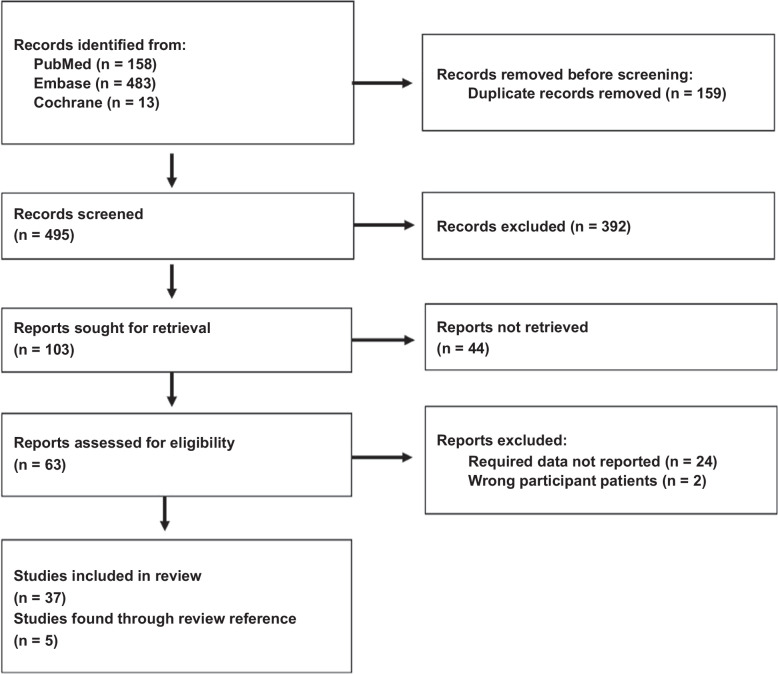
PRISMA flow diagram. Abbreviation: PRISMA, Preferred Reporting Items for Systematic Reviews and Meta-Analyses.

**FIGURE 2:**
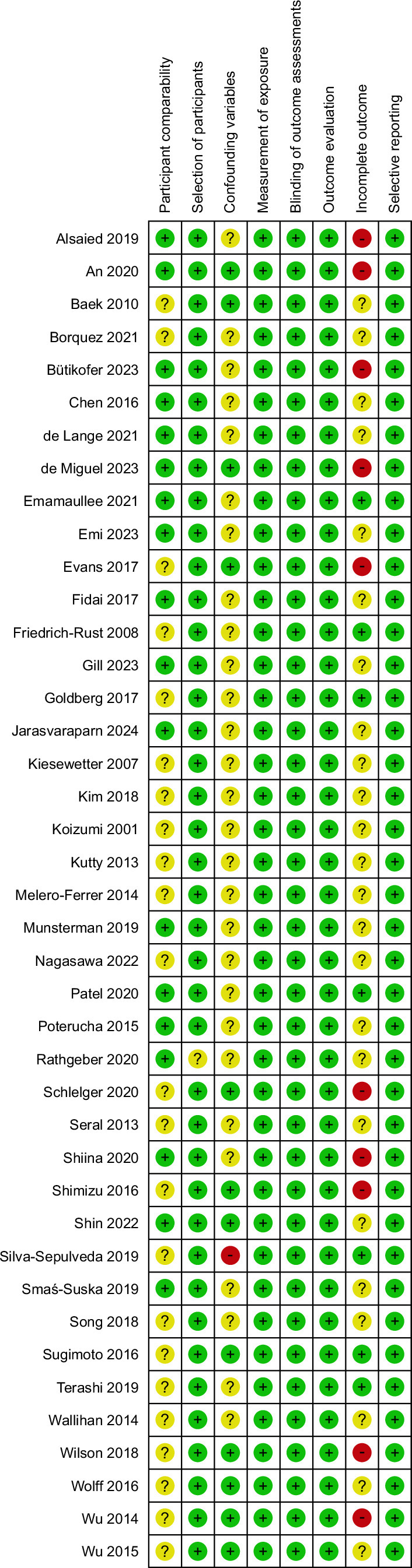
The summary of RoBANS assessment of the methodological quality of the studies. Abbreviation: RoBANS, risk of bias assessment tool for nonrandomized studies.

A total of 654 studies were identified from Embase (n=483), PubMed (n=158), Cochrane Library (n = 13), and a manual search (n=4). After removing 159 duplicates, a total of 495 studies remained.^3^,^11^,^12^,^13^,^14^,^15^,^16^,^17^,^18^,^19^,^20^,^21^,^22^,^23^,^24^,^25^,^26^,^27^,^28^,^29^,^30^,^31^,^32^,^33^,^34^,^35^,^36^,^37^,^38^,^39^,^40^,^41^,^42^,^43^,^44^,^45^,^46^ Of these, 392 were excluded for reasons such as case reports, editorials, letters, reviews, trial registers, notes, or titles and abstracts that were not aligned with our research. After excluding 44 articles due to a lack of original texts, 63 articles were evaluated. We excluded 24 studies that lacked relevant indicators, 1 with inappropriate subjects and 1 with high selection bias. The remaining 37 studies, along with 5 additional studies found through a manual review of references, were reviewed, resulting in a total of 42 studies. A flow diagram of the identification and selection process is presented in Figure 1. All 42 included studies were nonrandomized and observational. A summary of the quality evaluation results is presented in Figure 2.

### Studies using liver biopsy

A summary of studies that conducted liver biopsies is presented in Table 1. In the prospective studies by Kutty et al^12^ and Wu et al,^13^ liver biopsies were performed to validate imaging findings. In contrast, other studies conducted liver biopsies based on the clinical protocols for managing Fontan patients at each center or due to clinical signs and symptoms, with biopsies being performed as part of the clinical evaluation. These studies were evaluated retrospectively. We performed a meta-analysis of these studies, classifying liver fibrosis into mild (score ≤2) and severe (score 3–4) categories to identify factors associated with fibrosis severity. For the 4 studies that did not stratify severity, a meta-analysis was conducted on the correlation (Pearson correlation coefficient) between histological biopsy results and Fontan duration.

**TABLE 1:** Studies using liver biopsy to diagnose the FALD

References	Study	N (n)	Population	Age at Ex (y)^a^	Duration after Fontan op (y)^a^	Reason for liver bx	Scoring system	Anatomy	Systemic ventricle	Fontan type	Imaging modality	Correlation with histopathology
Kiesewetter et al^11^	Retro.	11	Both	24.6 ± 8	14.1 ± 5.0	Clinical evaluation	METAVIR Fibrosis Score	TA DILV	No mention	AP LT TCPC	CT	Fibrosis score ≤2 vs. >3 –zonal enchancement (n) 3/4 vs. 1/7, (*p* = 0.033) –reticular enhancement (n) 2/4 vs. 6/7 (*p* = 0.183)
Kutty et al^12^	Pros.	41	Both	13.8 ± 6.3	11 ± 6	To confirm the image	No mention	No mention	RV 39% LV 61%	LT 54% EC 46%	SWE	Fibrosis score <2 vs. ≥2 13.4 ± 1.3 vs. 19.8 ± 2.6 kPa, (*p* = 0.002)
Wu et al^13^	Pros.	50 (10)	Both	13.1 (2.4–57.7)	9.9 (0.1–32.5)	To confirm the image	METAVIR fibrosis staging	TA 18% DILV 16% HLHS 32% Heterotaxy 6%	RV 54% LV 46%	AP 8% LT 82% EC 8%	TE	1. Portal fibrosis score ≤2 vs. >3 34.8 (14.3–66.5) vs. 21.1 (16.8–22.6) kPa, (*p* = 0.14) 2. Central fibrosis score ≤2 vs. >3 14.3, 16.8 vs. 26.0 (21.1–66.5) kPa (*p* = 0.05)
Poterucha et al^14^	Retro.	50	Adults	25 (21–33)	22 (16–26)	Clinical evaluation	Ishak Scheuer Schwartz	TA 24% PA with IVS 2% DILV 32% DORV 16% DIRV 2% AVSD 12% Heterotaxy 12%	RV 40% LV 60%	AP 40% LT 34% EC 26%	MRE	Correlation coefficient^b^ *R* = 0.74 (*p* =0.02)
Wu et al^15^	Retro.	68	Both	23.2 (5.0–52.7)	18.1 (1.2–32.7)	Clinical evaluation	METAVIR fibrosis staging	TA 21% DILV 23% HLHS 10% Heterotaxy 19% others 26%	RV 37% LV 56% mix 7%	AP 38% LT 50% EC 6%		
Evans et al^16^	Retro.	30	Both	17 (6–45)	15 (1–29)	Clinical evaluation	Modified Scheuer staging and sinusoidal fibrosis staging	No mention	No mention	No mention	SWE	Correlation coefficient^b^ *R* = 0.6 (*p* = 0.002)
Goldberg et al^3^	Retro.	67	Both	17.3 ± 4.5	14.9 ± 4.5	Routine screening	Calculate quantitative collagen deposition by Leica Biosystems	No mention	RV 55% LV or mixed 44%	LT 60% EC 36%		
Munsterman et al^17^	Pros.	38	Adults	27 ± 6.6	21.4 ± 5.5	Routine screening	Fontan-specific fibrosis scores and collagen proportion area	TA and PA 47% DILV 29% DORV 5% HLHS 10% DORV 5% Heterotaxy 3%	No mention	AP 37% LT 18% EC 40%	TE	Fibrosis score ≤2 vs. >3 21.3 (14.3–29.1) vs. 26.0 (15.1–28.9) kPa (*p* = 0.511)
Silva-Sepulveda et al^18^	Retro	49	Both	17.8 (5–39)	15.2 (2–33)	Routine screening	Ishak fibrosis stage congestive hepatic fibrosis score and Modified Ishak congestive hepatic fibrosis	TA 18% HLHS 14% PA w IVS 12% DILV 12% DORV 10% AVSD 10% Heterotaxy 16% Others 6%	RV 39% LV 61%	AP 4% LT 59% EC 37%	MRE	Correlation coefficient^b^ *R* = 0.53 (*p =*0.004)
Patel et al^19^	Retro	57	Ped	14.6 ± 2.8	11.4 ± 2.9	Routine screening	Congestive Hepatic Fibrosis Score	No mention	RV 56% LV or Mixed 44%	EC 93%		
Emamaullee et al^20^	Retro	106	Ped	14.4 ± 3.5	10.8 ± 3.6	Routine screening	Congestive Hepatic Fibrosis Score	No mention	RV 52% LV 44.%	LT 6% EC 94%		
Borquez et al^21^	Retro	125	Both	15 (2–50.5)	12.7 (1−31)	Routine screening	Ishak fibrosis stage and Congestive hepatic fibrosis score and Modified Ishak congestive hepatic fibrosis	TA 32% Heterotaxy 14% HLHS 27% PA wIVS 14% DILV 17% DORV 8% AVSD 8% Others 6%	RV 51% LV 46%	No mention		
Shin et al^22^	Retro	45	Adults	25.9 ± 6.5	20.8 ± 4.8	Routine screening	Batts and Ludwig scoring system	No mention	RV 42.2%	AP 27% LT 49% EC 24%	TE	Fibrosis score ≤2 vs. >3 24.8 ± 20.6 vs. 23.3 ± 8.2 kPa (*p* = 0.85)
Bütikofer et al^23^	Pros.	50	Adults	25.9 (19.5–34.0)	21.8 (16.7–27.8)	Clinical evaluation	Congestive Hepatic Fibrosis Score	No mention	RV 20% LV 80%	AP 38% TCPC 62%	TE	Fibrosis score ≤2 vs. >3 23.4 (13.0–33.63) vs. 21.3 (17.3–26) kPa (*p* = 0.911)
Martin de Miguel et al^24^	Retro	159 (31)	Adults	31.5 ± 9.3	Not assessed	Clinical evaluation	Congestive Hepatic Fibrosis Score	TA 33% DILV 30% DORV 12% PA w IVS 11% HLHS 4% Heterotaxy 10% others 11%	RV 22%	AP 58% LT 20% EC 9%		
Jarasvaraparn et al^25^	Retro	66	Both	24.3 ± 9.3	20.3 ± 7.1	Routine screening and clinical evaluation	Congestive Hepatic Fibrosis Score	No mention	No mention	No mention	TE	Fibrosis score ≤2 vs. >3 25.66 ± 15.88 vs. 28.35 ± 15.84 kPa (*p* = 0.67)

^a^ Mean ± SD or median (range or IQR).

^b^ refer to the Pearson correlation coefficient

Abbreviations: AP, atriopulmonary; AVSD, atrioventricular septal defect; Bx, biopsy; DILV, double inlet left ventricle; DIRV, double inlet right ventricle; DORV, double outlet right ventricle; EC, extracardiac conduit; Ex, examination; IVS, intact ventricular septum; LT, lateral tunnel; LV, left ventricle; MRE, magnetic resonance elastography; N, total number of patients; n, number of cases that received a liver biopsy if not all patients underwent the testing; PA, pulmonary atresia; Ped, pediatrics; Prosp, prospective; Retro, retrospective; RV, right ventricle; SWE, shear wave elastography; TA, tricuspid atresia; TE, transient elastography.

### Hemodynamic and hematologic factors associated with FALD diagnosed by liver biopsy

**FIGURE 3:**
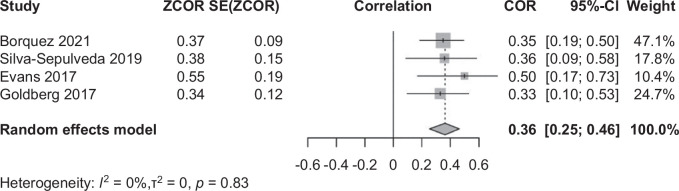
The correlation between the time since the Fontan operation and Fontan fibrosis confirmed by liver biopsy. The size of each square is proportional to the study’s weight. The diamond is the summary estimate. Abbreviation: COR, correlation coefficient.

The fibrosis scores from liver biopsies showed a weak positive correlation with the Fontan duration (*R* = 0.36, 95% CI: 0.25–0.46; Figure 3). In the subgroup analysis, there was no significant difference in the Fontan duration between the mild and severe liver fibrosis groups for children and adults, respectively. The pooled estimated Fontan duration was ~22.0 years for adults with severe fibrosis and 11.7 years for children (95% CI: 20.99–23.33 in adults and 95% CI: 11.29–12.71 in children; Table 2, Supplemental Figure S1, http://links.lww.com/HC9/B74).

**TABLE 2:** Comparison of patient characteristics and outcomes across studies in the meta-analysis

Parameter	Age group	No. studies	Total patients (mild/severe)	Summary weighted mean (mild)	Summary weighted mean (severe)	*p*	Total (random effect model, 95% CI)	Heterogeneity (*I*², *p*)
Fontan duration (year)	Adults	3	142 (45/97)	21.87	22.98	*0.84*	21.98 (20.66, 23.29)	71%, *0.03*
	Children	2	125 (75/50)	13.21	14.22	*0.44*	13.71(12.31, 15.12)	71%, *0.02*
Age at Fontan operation (y)	Adults	2	97 (36/61)	6.26	4.41	*0.12*	4.73 (3.82, 5.64)	27%, *0.25*
	Children	2	125 (75/50)	2.74	3.45	*0.41*	3.04 (2.24, 3.85)	96%, *<0.01*
Fontan pressure (mm Hg)	Adults and children	5	379 (183/196)	12.56	14.73	***0.03***	13.60 (12.43, 14.78)	80%, *<0.01*
Platelets (×10^3^/L)	Adults and children	5	305 (136/169)	189.24	166.60	***0.04***	176.61 (164.39, 188.84)	70%, *<0.01*
GGT (IU/L)	Adults and children	4	257 (141/116)	65.88	69.24	*0.60*	66.82 (61.15, 72.49)	0%, *0.43*
Total bilirubin (mmol/L)	Adults and children	4	257 (141/116)	0.93	1.13	*0.25*	1.01 (0.86, 1.15)	75%, *<0.01*
APRI	Adults and children	4	241 (119/122)	0.38	0.52	***<0.01***	0.45(0.38, 0.52)	75%, *<0.01*
Fib-4	Adults	2	88 (38/50)	0.77	1.32	***<0.01***	1.13(0.74, 1.51)	82%, *<0.01*
	Children	2	115 (75/40)	0.40	0.67	***0.03***	0.51(0.37, 0.65)	77%, *<0.01*
MELD-XI	Adults and children	3	210 (112/98)	10.09	10.83	*0.19*	10.27 (9.88, 10.66)	55%, *0.05*

Bold values indicate statistically significance *p* < 0.05.

Abbreviations: APRI, aspartate aminotransferase to platelet ratio index; Fib-4, Fibrosis-4 Index; MELD-XI, Model for End-Stage Liver Disease Excluding International Normalized Ratio.

Regarding the age at Fontan surgery, adults in the severe group were younger than those in the mild group, but this difference was not statistically significant. In children, high heterogeneity among studies prevented statistically significant differences in the severity of liver fibrosis (Table 2, Supplemental Figure S2, http://links.lww.com/HC9/B74). The pooled estimated age at Fontan operation was 4.7 years for adults (95% CI: 3.82–5.54) and 3.0 years for children (95% CI: 2.24–3.85).

Cardiac catheterization and blood tests to evaluate liver function were similarly performed based on the clinical protocols for managing Fontan patients at each center or due to clinical signs and symptoms. The Fontan pressure was generally higher in patients with severe liver fibrosis (*p* = 0.03) than in those with mild fibrosis, although substantial heterogeneity was observed (mild: *I*^2^ = 67%, severe: *I*^2^ = 71%) (Table 2, Supplemental Figure S3, http://links.lww.com/HC9/B74). The estimated pooled Fontan pressure was 12.6 mm Hg for mild fibrosis (95% CI: 11.33–13.78) and 14.7 mm Hg for severe fibrosis (95% CI: 13.14–16.33).

Subgroup analysis of biomarkers showed significant differences in platelet levels, aspartate aminotransferase to platelet ratio index (APRI), and fibrosis-4 index (Fib-4) between mild and severe liver fibrosis. However, there were no significant differences in other serological biomarkers, including the Model for End-Stage Liver Disease Excluding International Normalized Ratio (MELD-XI), GGT, bilirubin, and albumin, which are other biomarkers indicating liver function (Table 2, Supplemental Figure S4, http://links.lww.com/HC9/B74).

Despite 1 outlier study, severe liver disease was associated with lower platelet counts (*p*=0.04; Table 2, Supplemental Figure S, http://links.lww.com/HC9/B74). The pooled estimated platelet count was 189 × 10^3^/μL in the mild group (95% CI: 169.88–208.60) and 166 × 10^3^/μL in the severe group. Moderate heterogeneity was observed in the mild subgroup (mild: *I*^2^ = 69%, severe: *I*^2^ = 23%).

For analysis of APRI, the APRI was significantly elevated in the severe group compared to the mild group (*p* < 0.01; Table 2, Supplemental Figure S4B, http://links.lww.com/HC9/B74). The pooled estimated APRI value was ~0.45 (95% CI: 0.38–0.52), and moderate heterogeneity was observed in the studies in the mild group (mild: *I*^2^ = 65% and severe: *I*^2^ = 18%).

As the Fib-4 formula includes age (Fib-4 Score = [Age × aspartate aminotransferase]/[Platelets in 10^9^/L × √{alanine aminotransferase}]), meta-analyses were conducted separately for adults and children. Figure S4E shows that Fib-4 was significantly elevated in the severe group in both adults (*p <* 0.01) and children (*p* = 0.03). The pooled estimated Fib-4 was 1.13 (95% CI: 0.74–1.51) for adults and 0.51 (95% CI: 0.37–0.65) for children. There was moderate heterogeneity among the studies in the severe subgroup of adults (mild; *I*^2^ = 0%; and severe; *I*^2^ = 62%), whereas heterogeneity was lower in children (mild; *I*^2^ = 0% and severe; *I*^2^ = 38%).

### Correlation between noninvasive imaging tests and histologic evaluation

The outcomes of studies on the correlation between liver fibrosis confirmed by liver biopsy and noninvasive imaging modalities are summarized in Table 1. Many studies have found significant correlations between the histological severity of liver fibrosis and both magnetic resonance elastography (MRE) and shear wave elastography, with notable differences between mild and severe fibrosis groups. However, no significant differences in liver stiffness measured by transient elastography were observed between the groups. Owing to insufficient studies, correlations for liver conditions using CT and MRI could not be confirmed.

### Clinical parameters associated with liver fibrosis

The outcomes of the studies reporting hemodynamic parameters from cardiac catheterization are summarized in Supplemental Table S3, http://links.lww.com/HC9/B74. Despite differences between diagnostic modalities, many studies have found significant correlations between liver fibrosis and hemodynamic parameters such as Fontan pressure, CVP, and left ventricular end-diastolic pressure, or meaningful differences in outcomes between mild and severe fibrosis. In particular, studies using MRE showed significant correlations with hemodynamic parameters such as Fontan pressure, CVP, and cardiac index, especially when compared to other imaging modalities.

A summary of the studies that analyzed factors associated with liver fibrosis is presented in Supplemental Table S4, http://links.lww.com/HC9/B74. Although the results varied across studies, several studies revealed a significant correlation between biochemical factors, such as GGT, platelet count, APRI, Fib-4, and MELD-XI, and liver fibrosis diagnosed using liver biopsy or noninvasive imaging modalities. The severe liver fibrosis group tended to have lower platelet counts, higher APRI scores, and higher Fib-4 levels than those in the mild liver fibrosis group.

## DISCUSSION

Patients with persistent Fontan physiology, characterized by elevated CVP and reduced cardiac output, are at risk of multiorgan failure, including liver disease. Although studies on the incidence of FALD are limited, evidence suggests that its prevalence increases with time post-Fontan surgery. According to a study by Pundi et al,^47^ liver cirrhosis occurred in approximately half of the 195 patients 30 years after the Fontan operation, although not all cases were histologically verified. Recently, Hitawala et al,^48^ not included in our review, reported a cumulative incidence of liver cirrhosis of 1.61 cases per 100 person-years and 32.2% at 20 years after Fontan surgery.

While the leading cause of death in patients with Fontan circulation remains unclear, FALD is increasingly recognized as a significant contributor to morbidity and mortality. A national study in Japan reported a 17.8% mortality rate due to liver disease in Fontan patients. In addition, a study in Quebec found that severe FALD tripled mortality risk.^49^ Early prediction, diagnosis, and management of FALD are crucial strategies for reducing liver disease–related mortality in Fontan patients. Despite its limitations, liver biopsy remains the gold standard for diagnosing FALD. Nevertheless, research into noninvasive methods for diagnosing FALD is essential for developing protocols to monitor complications in Fontan patients.

Our review found a weak correlation between liver fibrosis and Fontan duration, with no significant difference in post-Fontan fibrosis severity. In addition, the mean age at the time of the Fontan procedure did not predict advanced fibrosis. Aligning with Hitawala et al,^48^ who noted a rapid increase in liver cirrhosis 20 years after Fontan, our meta-analysis confirmed an average of 22 years from Fontan to fibrosis diagnosis. Despite the lack of studies with serial liver biopsies in the same cohort to predict the onset of severe liver fibrosis, a 20-year postoperative inflection point for histological liver changes is reasonable.

In our analysis, severe liver fibrosis developed ~12 years after the Fontan operation in pediatric patients. This suggests that additional risk factors, such as underlying congenital cardiac defects and preoperative characteristics (eg, birth history, genetics, and growth status), may contribute to early liver disease following the Fontan procedure. Due to limited data on liver disease evaluation before the Fontan operation, it is unclear whether patients with early liver disease had preexisting fibrosis. However, several studies, including those by Johnson et al^5^ and Schwartz et al,^6^ have suggested that liver injury may occur before the Fontan operation and that further injury may begin soon after the operation. They suggested that liver injury may occur before Fontan due to hypoxia-ischemia and high right atrial pressure and that further injury may begin soon after the operation.

Various methods are used to assess FALD severity, including clinical evaluations, biochemical/hematological parameters, noninvasive fibrosis scores, radiographic imaging, elastography, and invasive liver histology. However, findings regarding the severity of FALD in these studies are diverse.^14^,^31^,^41^ Despite limited studies, our review found that MRE and shear wave elastography measurements correlated with liver biopsy results, while transient elastography did not. This discrepancy may be due to the influence of factors such as obesity and ascites. A previous study on MRE reproducibility and reliability reported favorable sensitivity and specificity for detecting the differences between early-stage and late-stage fibrosis.^32^ Another study found a correlation between clinical deterioration (including death and heart transplantation) and liver stiffness measured using MRE in patients with Fontan circulation.^50^ However, liver stiffness increases in both liver fibrosis and congestive hepatopathy, making it difficult to distinguish whether stiffness in patients with FALD is due to fibrosis or congestion.^4^ To distinguish between liver congestion and fibrosis, it is necessary to comprehensively evaluate and understand additional factors that can complement liver stiffness measurements obtained through elastography. In addition, although our analysis did not show meaningful results for CT and MRI, this was likely due to the limited number of studies available for these modalities, which presented a limitation in our analysis. Therefore, further research on CT, MRI, and other new noninvasive methods is needed.

Our meta-analysis found significantly higher Fontan pressure in patients with severe liver fibrosis compared to those with mild fibrosis. However, as suggested in a previous study,^6^ because the pressure remained within the optimal range (<15 mm Hg), the severity of FALD could not be reliably predicted by the absolute level of Fontan pressure alone. Acceptable Fontan hemodynamics does not preclude liver fibrosis; thus, monitoring Fontan pressure trends is essential.

Biochemical markers showed significant differences: lower platelet counts and higher APRI and Fib-4 values in severe fibrosis cases, although these values were still within the normal limits (<0.5 APRI, <1.3 Fib-4). Therefore, the cutoff levels for other types of hepatitis cannot be applied to Fontan patients, emphasizing the need to monitor these markers over time. Due to the age factor included in the calculation of Fib-4, where the age range in children is small and the marker lacks validation in pediatric populations, Fib-4 is not a reliable marker in children. Further research is needed to determine the effectiveness of Fib-4 as a marker of fibrosis.

Our review is meaningful because it attempted to classify mild and severe liver fibrosis, distinguish it from mild liver fibrosis that may have occurred at any time, and identify factors associated with severe liver fibrosis. Evaluating the severity of FALD is crucial for tracking disease progression. This evaluation helps ensure appropriate management and identify patients who may benefit from liver transplantation, particularly in the context of combined heart-liver transplantation, which is becoming increasingly prevalent. Our review suggests that routine monitoring for liver disease should begin at least 10 years after Fontan surgery. In our review, MRE showed a meaningful correlation with histologic findings and hemodynamic factors, such as Fontan pressure; however, the optimal methods for diagnosing FALD remain controversial. Further research is needed to clarify the diagnostic usefulness of MRE, considering its cost and availability.

Our study had several limitations. The reliance on mostly retrospective data may introduce selection bias, as liver biopsies were likely performed only in symptomatic or suspected patients, possibly underestimating the true prevalence and severity of liver fibrosis. In addition, the lack of patient-level data prevented us from considering individual baseline characteristics, such as age and anatomical factors. The inherent heterogeneity in the included studies, including variability in study designs and patient populations, may have contributed to the high heterogeneity observed in our results, potentially limiting the generalizability of our conclusions. Furthermore, due to the observational nature of the studies, none of the included studies were designed to compare outcomes between the Fontan patient population and a control group, limiting our ability to establish causality. Future research should aim to account for confounding factors within the population to identify more objective risk factors for severe fibrosis. In addition, given the correlation between Fontan duration and liver fibrosis, further studies are needed to identify other significant risk factors for severe fibrosis, particularly in children with early-onset disease.

In conclusion, advances in Fontan surgery have increased the chances of survival in adulthood. However, long-term follow-up revealed that liver complications, such as fibrosis, cirrhosis, and HCC, substantially increase the morbidity and mortality of patients with Fontan circulation. Because FALD is preventable and treatable to a certain extent, early prediction is crucial. Liver fibrosis, as assessed by liver biopsy, demonstrated a positive correlation with the duration of Fontan circulation. Biochemical factors, such as the platelet count, APRI, and Fib-4 levels, along with hemodynamic factors, such as Fontan pressure, predicted the severity of Fontan fibrosis. Monitoring trends in these values is essential for timely intervention. MRE was found to be more accurate than other imaging modalities in delineating liver fibrosis. However, further research is required to determine the reliability of MRE as an alternative to liver biopsy in evaluating FALD in patients after the Fontan procedure.

## Supplementary Material

SUPPLEMENTARY MATERIAL
