# Supplementary material for: Clinical predictors and noninvasive imaging in Fontan-associated liver disease: A systematic review and meta-analysis
Source: Hepatol Commun. 2024 Nov 15;8(12):e0580. doi: 10.1097/HC9.0000000000000580 (PMC11567714; doi:10.1097/HC9.0000000000000580)
Supplement: SUPPLEMENTARY MATERIAL [file hc9-8-e0580-s001.pdf]

Clinical Predictors and Non-Invasive Imaging in Fontan-Associated Liver Disease: A Systematic Review and Meta-Analysis

Short Title: Liver Biopsy Versus Imaging in FALD

Jae Hee Seol[1,2], Jin Young Song[4], Su Jin Kim[5], Hoon Ko[6], Jae Yoon Na[7], Min Jung Cho[8], Hee Jung Choi[9], Jue Seong Lee[10], Kyung Jin Oh[11], Jo Won Jung[*3], Se Yong Jung[*3]

[1.] Department of Pediatrics, Yonsei University Wonju College of Medicine, Wonju, Republic of Korea

[2.] Department of Pediatrics, Yonsei University College of Medicine, Seoul, Republic of Korea

[3.] Division of Pediatric Cardiology, Department of Pediatrics, Congenital Heart Disease Center, Severance Cardiovascular Hospital, Yonsei University College of Medicine, Seoul, Republic of Korea

[4.] Department of Pediatrics, Samsung Medical Center, Sungkyunkwan University School of Medicine, Seoul, Republic of Korea

[5.] Department of Pediatrics, Sejong General Hospital, Buchun, Republic of Korea

[6.] Department of Pediatrics, Pusan National University Yangsan Hospital, Pusan National University School of Medicine, Yangsan, Republic of Korea

[7.] Department of Pediatrics, Hanyang University College of Medicine, Seoul, Republic of Korea

[8.] Department of Pediatrics, College of Medicine, Gyeoungsang National University Changwon Hospital, Changwon, Geongsangnam-do, Republic of Korea

[9.] Department of Pediatrics, Keimyung University Dongsan Hospital, Keimyung University School of Medicine, Daegu, Republic of Korea

[10.] Department of Pediatrics, Korea University Anam Hospital, Korea University College of Medicine, Seoul, Republic of Korea

[11.] Department of Pediatrics, Seoul Metropolitan Government-Seoul National University Boramae Medical Center,

Seoul, Republic of Korea

Abbreviations: APRI, aspartate aminotransferase to platelet ratio index; CI, confidence interval; CT, computed tomography; CVP, central venous pressure; FALD, Fontan-associated liver disease; FIB-4, fibrosis-4 index; GGT, gamma-glutamyl transferase; INR, international normalized ratio; MELD-XI, Model for End-Stage Liver Disease Excluding International Normalized Ratio; MRE, magnetic resonance elastography; MRI, magnetic resonance imaging; RoBANS, risk of bias assessment tool for nonrandomized studies; SWE, shear wave elastography; TA, tricuspid atresia; TE, transient elastography; US, ultrasound

Correspondence:

Jung Jo-won, jwjung@yuhs.ac

Jung Se-young, jung811111@yuhs.ac

Supplementary Table 1: Search Strategies

Database: PubMed
Platform: US National Library of Medicine
Date Searched: February 10, 2024 / **Date of Search Update:** April 10, 2024
Other Limits/Filters: Exclude publication types: editorial, commentary, protocol, case report, case series, reviews, letter

Set	Concept	Search Strategy
#1	Fontan	Fontan Procedure[mh] OR Fontan[tw]
#2	Singe ventricle heart	Univentricular Heart/surgery[mh] OR univentricular heart*[tw] OR single ventric*[tw] OR hypoplastic left heart syndrome*[tw] OR left heart hypoplasia syndrome*[tw]
#3		#1 OR #2
#4	Liver disease	Liver Diseases[mh] OR liver disease*[tw] OR liver disorder*[tw] OR liver dysfunction*[tw] OR hepatic disease*[tw] OR hepatic disorder*[tw] OR liver cirrhosis[tw] OR hepatic cirrhosis[tw] OR cardiac cirrhosis[tw] OR hepatocellular carcinoma*[tw] OR hepato-cellular carcinoma*[tw] OR liver cell carcinoma*[tw] OR hepatic cell carcinoma*[tw] OR adult liver cancer*[tw] OR hepatoma*[tw]
#5		#3 AND #4
#6	US	Ultrasonography[mh] OR ultrasonograph*[tw] OR ultrasound[tw] OR ultrasonic*[tw]
#7	Elastography	Elasticity Imaging Techniques[mh] OR elasticit*[tw] OR elastograph*[tw] OR vibro-acoustograph*[tw] OR sonoelastograph*[tw] OR elastogram*[tw] OR acoustic radiation force impulse[tw] OR ARFI imaging[tw]
#8	MRI	Magnetic Resonance Imaging[mh] OR magnetic resonance[tw] OR MR imaging[tw] OR MRI[tw]
#9	CT	Tomography, X-Ray Computed[mh] OR computed tomograph*[tw] OR CT scan*[tw] OR computer assisted tomograph*[tw] OR computerized tomograph*[tw]
#10		#6 OR #7 OR #8 OR #9
#11		#5 AND #10

#12	Limits & Filters	(Animals[mh] NOT Humans[mh]) OR Models, Animal[mh:noexp] OR Disease Models, Animal[mh] OR Animal Experimentation[mh]
#13	Limits & Filters	Case Reports[pt] OR case stud*[tw] OR case report*[tw] OR case series[tw] OR ((cases[tw] OR case[tw]) NOT (case control*[tw] OR case match*[tw] OR case compar*[tw]))
#14		#11 NOT #12 NOT #13

Notes: The limits for language (English) and publication year (1971–2023) were applied to the main search using the filters available in PubMed. The keywords were searched in the title field only (i.e., [Title]), title and abstract fields in PubMed (i.e., [Title/Abstract]), publication type field (i.e. [Publication Type]), and the controlled vocabulary terms are indicated with [Mesh]. Phrases were enclosed in quotation marks to force the searching of the exact terms in order presented. To these results, the search strategy to exclude publication types was applied.

Database: Embase
Platform: Elsevier
Date Searched: February 10, 2024 / **Date of Search Update:** April 10, 2024
Date Limits: 1971 – 2023 & 2023 – 2023;
Other Limits/Filters: Source: Embase & Embase Classic; Language: English; Exclude publication types: editorial, commentary, protocol, case report, case series, reviews, letter

Set	Concept	Search Strategy
#1	Fontan	'fontan procedure'/exp OR 'fontan procedure' OR fontan:ti,ab,kw
#2	Single ventricle heart	'heart single ventricle'/dm_su OR 'univentricular heart*':ti,ab,kw OR 'single ventric*':ti,ab,kw OR 'hypoplastic left heart syndrome*':ti,ab,kw OR 'left heart hypoplasia syndrome*':ti,ab,kw
#3		#1 OR #2
#4	Liver disease	'liver disease'/exp OR (((liver OR hepatic) NEXT/1 (disease* OR disorder* OR dysfunction* OR cirrhosis)):ti,ab,kw) OR 'cardiac cirrhosis':ti,ab,kw OR 'adult liver cancer*':ti,ab,kw OR hepatoma*:ti,ab,kw OR (((hepatocellular OR 'hepato cellular' OR liver OR hepatic) NEXT/2 carcinoma*):ti,ab,kw)
#5		#3 AND #4
	US	'echograph'/exp OR ultrasonograph*:ti,ab,kw OR echograph*:ti,ab,kw OR ultrasound:ti,ab,kw OR ultrasonic*:ti,ab,kw
	Elastography	'echograph'/exp OR 'elastography'/exp OR elasticit*:ti,ab,kw OR elastograph*:ti,ab,kw OR 'vibro acoustograph*':ti,ab,kw OR sonoelastograph*:ti,ab,kw OR elastogram*:ti,ab,kw OR 'acoustic radiation force impulse':ti,ab,kw OR arfi:ti,ab,kw
	MRI	'echograph'/exp OR 'elastography'/exp OR 'nuclear magnetic resonance imaging'/exp OR 'magnetic resonance':ti,ab,kw OR 'mr imaging':ti,ab,kw OR mri:ti,ab,kw

	CT	'echograph'/exp OR 'elastography'/exp OR 'nuclear magnetic resonance imaging'/exp OR 'x-ray computed tomography'/exp OR 'computed tomograph*':ti,ab,kw OR 'ct scan*':ti,ab,kw OR 'computer assisted tomograph*':ti,ab,kw OR 'computerized tomograph*':ti,ab,kw
		#6 OR #7 OR #8 OR #9
		#5 AND #10
	Limits & Filters	'animal'/exp NOT 'human'/exp OR 'animal model'/exp OR 'animal experiment'/exp OR [animal cell]/lim OR [animal experiment]/lim OR [animal model]/lim OR [animal tissue]/lim
	Limits & Filters	'case study'/de OR 'case report'/de
		#11 NOT #12 NOT #13
	Limits & Filters	#14 AND ('conference abstract'/it OR 'conference paper'/it OR 'conference review'/it)
		#14 NOT #15

Database: Cochrane
Platform: EBSCOhost
Date Searched: : February 10, 2024 / **Date of Search Update:** April 10, 2024
Date Limits: 1971 – 2023 & 2023 – 2023;
Other Limits/Filters: Source: Embase & Embase Classic; Language: English; Exclude publication types: editorial, commentary, protocol, case report, case series, reviews, letter

Set	Concept	Search Strategy
#1	Fontan	[mh "Fontan Procedure"] OR Fontan:ti,ab,kw
#2	Single ventricle heart	[mh "Univentricular Heart"] OR (univentricular NEXT heart*):ti,ab,kw OR (single NEXT ventric*):ti,ab,kw OR ("hypoplastic left heart" NEXT syndrome*):ti,ab,kw OR ("left heart hypoplasia" NEXT syndrome*):ti,ab,kw
#3		#1 OR #2
#4	Liver disease	[mh "Liver Diseases"] OR ((liver OR hepatic) NEXT/1 (disease* OR disorder* OR dysfunction* OR cirrhosis)):ti,ab,kw OR ("cardiac cirrhosis" OR hepatoma*):ti,ab,kw OR (adult NEXT liver NEXT cancer*):ti,ab,kw OR ((hepatocellular OR hepato-cellular OR liver OR hepatic) NEXT/2 carcinoma*):ti,ab,kw
#5		#3 AND #4

Supplementary Table 2: PRISMA check list

Section and Topic	Item #	Checklist item	Location where item is reported
**TITLE**			
Title	1	Identify the report as a systematic review.	Title
**ABSTRACT**			
Abstract	2	See the PRISMA 2020 for Abstracts checklist.	Abstract
**INTRODUCTION**			
Rationale	3	Describe the rationale for the review in the context of existing knowledge.	Introduction
Objectives	4	Provide an explicit statement of the objective(s) or question(s) the review addresses.	Introduction
**METHODS**			
Eligibility criteria	5	Specify the inclusion and exclusion criteria for the review and how studies were grouped for the syntheses.	Methods
Information sources	6	Specify all databases, registers, websites, organisations, reference lists and other sources searched or consulted to identify studies. Specify the date when each source was last searched or consulted.	Methods
Search strategy	7	Present the full search strategies for all databases, registers and websites, including any filters and limits used.	Methods<br>Supplementary table 1
Selection process	8	Specify the methods used to decide whether a study met the inclusion criteria of the review, including how many reviewers screened each record and each report retrieved, whether they worked independently, and if applicable, details of automation tools used in the process.	Methods
Data collection process	9	Specify the methods used to collect data from reports, including how many reviewers collected data from each report, whether they worked independently, any processes for obtaining or confirming data from study investigators, and if applicable, details of automation tools used in the process.	Methods
Data items	10a	List and define all outcomes for which data were sought. Specify whether all results that were compatible with each outcome domain in each study were sought (e.g. for all measures, time points, analyses), and if not, the methods used to decide which results to collect.	Methods
	10b	List and define all other variables for which data were sought (e.g. participant and intervention characteristics, funding sources). Describe any assumptions made about any missing or unclear information.	Methods<br>Funding; end of page
Study risk of bias assessment	11	Specify the methods used to assess risk of bias in the included studies, including details of the tool(s) used, how many reviewers assessed each study and whether they worked independently, and if applicable, details of automation tools used in the process.	Methods.<br>Supplementary Figure 1
Effect measures	12	Specify for each outcome the effect measure(s) (e.g. risk ratio, mean difference) used in the synthesis or presentation of results.	results
Synthesis	13a	Describe the processes used to decide which studies were eligible for each synthesis (e.g. tabulating the study intervention characteristics	Methods

Section and Topic	Item #	Checklist item	Location where item is reported
methods		and comparing against the planned groups for each synthesis (item #5)).	
	13b	Describe any methods required to prepare the data for presentation or synthesis, such as handling of missing summary statistics, or data conversions.	Methods
	13c	Describe any methods used to tabulate or visually display results of individual studies and syntheses.	Methods
	13d	Describe any methods used to synthesize results and provide a rationale for the choice(s). If meta-analysis was performed, describe the model(s), method(s) to identify the presence and extent of statistical heterogeneity, and software package(s) used.	Methods
	13e	Describe any methods used to explore possible causes of heterogeneity among study results (e.g. subgroup analysis, meta-regression).	Methods
	13f	Describe any sensitivity analyses conducted to assess robustness of the synthesized results.	Methods
Reporting bias assessment	14	Describe any methods used to assess risk of bias due to missing results in a synthesis (arising from reporting biases).	Methods. Supplementary Figure 1
Certainty assessment	15	Describe any methods used to assess certainty (or confidence) in the body of evidence for an outcome.	Not conducted
**RESULTS**			
Study selection	16a	Describe the results of the search and selection process, from the number of records identified in the search to the number of studies included in the review, ideally using a flow diagram.	Results
	16b	Cite studies that might appear to meet the inclusion criteria, but which were excluded, and explain why they were excluded.	Results
Study characteristics	17	Cite each included study and present its characteristics.	Results
Risk of bias in studies	18	Present assessments of risk of bias for each included study.	Methods. Supplementary Figure 1
Results of individual studies	19	For all outcomes, present, for each study: (a) summary statistics for each group (where appropriate) and (b) an effect estimate and its precision (e.g. confidence/credible interval), ideally using structured tables or plots.	Results
Results of syntheses	20a	For each synthesis, briefly summarise the characteristics and risk of bias among contributing studies.	Results
	20b	Present results of all statistical syntheses conducted. If meta-analysis was done, present for each the summary estimate and its precision (e.g. confidence/credible interval) and measures of statistical heterogeneity. If comparing groups, describe the direction of the effect.	Results
	20c	Present results of all investigations of possible causes of heterogeneity among study results.	Results
	20d	Present results of all sensitivity analyses conducted to assess the robustness of the synthesized results.	Not conducted
Reporting biases	21	Present assessments of risk of bias due to missing results (arising from reporting biases) for each synthesis assessed.	Not conducted due to the inherent

Section and Topic	Item #	Checklist item	Location where item is reported
			limitation of non-RCT studies
Certainty of evidence	22	Present assessments of certainty (or confidence) in the body of evidence for each outcome assessed.	Not conducted
**DISCUSSION**			
Discussion	23a	Provide a general interpretation of the results in the context of other evidence.	Discussion
	23b	Discuss any limitations of the evidence included in the review.	Discussion
	23c	Discuss any limitations of the review processes used.	Discussion
	23d	Discuss implications of the results for practice, policy, and future research.	Discussion
**OTHER INFORMATION**			
Registration and protocol	24a	Provide registration information for the review, including register name and registration number, or state that the review was not registered.	methods
	24b	Indicate where the review protocol can be accessed, or state that a protocol was not prepared.	methods
	24c	Describe and explain any amendments to information provided at registration or in the protocol.	methods
Support	25	Describe sources of financial or non-financial support for the review, and the role of the funders or sponsors in the review.	End of page
Competing interests	26	Declare any competing interests of review authors.	End of page
Availability of data, code and other materials	27	Report which of the following are publicly available and where they can be found: template data collection forms; data extracted from included studies; data used for all analyses; analytic code; any other materials used in the review.	End of page

Supplementary Table 3: Results of the association between hemodynamic parameters and liver fibrosis severity noted by imaging or liver Biopsy

Reference	Study	N (n)	Popu-lation	Age at Ex(yr)*	Duration after Fontan op(yr)*	Modality	Hemodynamic parameters†					
							**Fontan Pr** (mmHg)	**CVP** (mmHg)	**PVRI** (WU/m2)	**CI** (L/min/m2)	**PAWP** (mmHg)	**LVEDP** (mmHg)
Silva-Sepulveda [9] 2019	Retro.	49	both	17.8 (5-39)	15.2 (2-33)	Bx	R=0.36, p=0.01		R=−0.152, p=0.30			R= 0.33, p=0.03
Borquez [22] 2021	Retro.	125	both	15 (2–50.5)	12.7 (1−31)	Bx	r=0.25, p<0.01					
Evans [17] 2017	Retro.	30	both	17 (6–45)	15 (1-29)	Bx			R=0.1, p=0.76			R=-0.1 p=0.74
Kiesewetter [6] 2007	Retro.	11	both	24.6 ±8	14.1±5.0	Bx	mild vs severe 11±2 vs 16±6 p=0.14					mild vs severe 6±2 vs 10±5, p=0.23
Wu [16] 2015	Retro.	68	both	23.2 (5.0–52.7)	18.1 (1.2–32.7)	Bx		mild vs severe 17±4 vs 17±4 p=0.72		mild vs severe 3±1vs 3±1, p=0.18	mild vs severe 12±4 vs 13±6 p=0.46	
Emamaullee [21] 2021	Retro.	106	Ped	14.4 ± 3.5	10.8 ± 3.6	Bx	mild vs severe 12±2 vs 14±4 p=0.23		mild vs severe 2±1 vs 2±1 p=0.35	mild vs severe 3±1 vs 3±2 p=0.37		mild vs severe 7±2 vs 8±4 p=0.28
Shin [23] 2022	Retro.	45	adults	25.9 ± 6.5	20.8 ± 4.8	Bx	mild vs severe 15±3 vs 14±3 p=0.46					mild vs severe 9±3 vs 12±3 p=0.04
Bütikofer [24] 2023	Pros.	97 (50)	adults	25.9 (19.5-34.0)	21.8 (16.7-27.8)	Bx	mild vs severe 11±3 vs 13±5 p=0.055					

Jarasvaraparn [26] 2024	Retro.	66 (47)	both	24.3 ± 9.3	20.3 ± 7.1	Bx	mild vs severe 13±3 vs 16±5, p=0.01<br><br>r=0.43, p<0.01					
Shimizu [27] 2016	Retro.	57	both	23.3 ± 9.9	16.2±5.4	CT		no LC vs LC 13±2 vs 14±5 p=0.02		no LC vs LC 2±1vs 2±1, =0.64		
Nagasawa [28] 2022	Retro.	27	both	22.1± 9.4	18.3 ± 8.2	CT		mild vs severe 11±3 vs 13±3 p=0.06				
Song [29] 2018	Retro.	26 (19)	both	13 (10.0-35.0)	10.5 (4-17)	CT			CPLD vs LC 1±1 vs 2±1, p<0.05		CPLD vs LC 12±2 vs 15±3, p<0.05	CPLD vs LC 10±4 vs 12±2 p=0.15
Shiina [30] 2020	Pros.	16	adults	31.3 ± 8.5	Not assessed	MRI		r=0.14, p=0.62	r=-0.08, p=0.79			
De Lange [31] 2021	cross-sectional	27	both	22.1± 9.4	18.3 ± 8.2	MRI		r=0.5, P<0.01				r=0.2, p=0.45
Wallihan [32] 2014	Retro.	14	both	18.2 (9.1-45.9)	16.5 (6.9-32.9)	MRE				R=-0.60 p=0.02		
Sugimoto [33] 2016	Pros.	16	both	15.3 (6.5–30.4)	12.9 (4.4–23.3)	MRE		r=0.8 p<0.01		r=-0.09, p=0.42		
Poterucha [15] 2015	Retro.	50 (30)	adults	25 (21-33)	22 (16-26)	MRE	R=0.41, p=0.03		R=0.53, p<0.01	R=0.43, p=0.02		
Silva-Sepulveda [19] 2019	Retro.	49 (28)	both	17.8 (5-39)	15.2 (2-33)	MRE	R=0.59, p<0.01		R=0.21, p=0.31	R=0.09, p=0.65		R=0.15, p=0.47
Alsaied [34] 2019	Retro.	70 (46)	adults	24.7(21.6-32.1)	17.9 (15.1-23.4)	MRE	r=0.34, p=0.03					r=-0.54, p=0.02
Koizumi [35] 2001	Pros.	43	both	17.0 (12.0–25.0)	15.3 (9.7–21.7)	SWE	r=0.20, p=0.24			r=-0.15, p=0.39	r=0.26, p=0.14	

Kim[36] 2018	Retro.	64	both	17.6 ± 5.3	12.1 ± 4.0	SWE		R=0.34, p<0.01	R=0.03 p=0.83			R=0.33, p<0.01
Terashi [37] 2019	cross-sectional	79	Ped	10.3 ± 4.9	Not assessed	SWE		R=0.78 p<0.00		R=0.45 p<0.001		
De Lange [31] 2021	Pros.	43/45	Ped	16.5 (15.4-17.9)	13.8 ± 2.9	SWE		r=0.2, p=0.3				r=0.03, p=0.9
Nagasawa [28] 2022	cross-sectional	27	both	22.1 ± 9.4	18.3 ± 8.2	SWE		r=0.31, p=0.16				
Kutty [13] 2013	Pros.	41 (16)	both	13.8 ± 6.3	11 ± 6	SWE			T=1.64, p=0.12	T=0.13, p=0.90	T=3.01, p=0.09	T=4.29, p=0.001
Koizumi [35] 2001	Pros.	43	both	17.0 (12.0–25.0)	15.3 (9.7–21.7)	TE	r=0.3 p=0.08			r=-0.02, p=0.90	r=0.06, p=0.74	
Wu [14] 2014	Retro. &Pros.	50 (49)	both	13.1 (2.4–57.7)	9.9 (0.1–32.5)	TE	R=0.31, p=0.04		R=0.34, p=0.03	R=-0.33, p=0.03	R=0.18, p=0.24	
Chen [38] 2016	Pros.	22		13.7 (5.9–16.8)	9.6 (1.0–12.9)	TE	R= 0.35, p = 0.11					
Rathgeber [39] 2020	Pros.	76	Ped	11.7 (8.4–14.8)	8.4 (4.6–11.4)	TE	r=0.32, p=0.3					
Shin [23] 2022	Retro.	45	adults	25.9 ± 6.5	20.8 ± 4.8	TE	r=0.2, p=0.34					r=-0.05, p=0.99
Bütikofer [24] 2023	Pros.	97 (94)	adults	23.1 (18.7-30.6)	18.3 (15.3-25.8)	TE	R=0.36, p=0.01					
Emi [40] 2023	Retro.	24	adults	35 (25–39)	27 (19–33)	TE		r=0.83, p<0.001				

* mean ± SD or median (range or interquartile range).

†Statistics : R, Pearson correlation coeffient; r, Spearman's rank correlation; T, linear regression

CI, cardiac index; CT, computerized tomography; Ex, examination; MRE, magnetic resonance elastography; CVP, central venous pressure; LVEDP, left ventricular end-diastolic pressure; MRI, magnetic resonance imaging; N, total number of patients; n, number of cases underwent testing; PAWP, pulmonary arterial wedge pressure; Ped, pediatrics; Prosp, prospective; PVRI, pulmonary vascular resistance index; Retro, retrospective; SWE, shear wave elastography; TE, transient elastography

Supplementary Table 4: Results of the association between biochemical parameters and liver fibrosis severity noted by imaging or liver Biopsy.

Reference	Study	N (n)	Popu-lation	Age at Ex(yr)*	Duration after Fontan op (yr)*	Modality	Biochemical parameters					
							**AST** Mild / Severe IU/L	**ALT** Mild / Severe IU/L	**ALP** Mild / Severe IU/L	**GGT** Mild / Severe IU/L	**Bil** Mild / Severe mg/dL	**Albu.** Mild / Severe g/L
Wu [16] 2015	Retro.	68	both	23.2 (5.0–52.7)	18.1 (1.2–32.7)	Bx	30(25-38) vs 33(26-42) p=0.47	25(17-34) vs 27(20-51) p=0.33	92(72-116) vs 101(89-188) p=0.04	70 (46–96) vs 76(53-132) p=0.52	1.0(0.5-1.3) vs 0.9(0.6–1.6), p=0.73	4.1(3.7-4.4) vs 4.0(3.3-4.4) p=0.46
Munsterman [18] 2019	Pros.	38	adults	27 ± 6.6	21.4 ± 5.5	Bx	28(23-33) vs 28(25-35) p=0.551	28(24-33) vs 28(23-37) p=0.915	70(54-97) vs 81(70-96) p=0.105	58(46-104) vs 62(49-121) p=0.636	0.8(0.6-1.1) vs 0.9 (0.6-1.3) p=0.761	41(40-43) vs 42(40-44) p=0.529
Emamaullee [21] 2021	Retro.	106	Ped	14.4 ± 3.5	10.8 ± 3.6	Bx	33 ± 8.4 vs 37±12.5, p=0.17	35±9.7 vs 36±12.5, p=0.97		64(37-82) vs 57(35-89) p=0.98	0.9 ± 0.7 vs 1.7±2.2 p<0.01	37 ± 6 vs 38 ± 6 p=0.11
Shin [23] 2022	Retro.	45	adults	25.9 ± 6.5	20.8 ± 4.8	Bx	22±5 vs 27±10, p=0.67	24±10 vs 26±15, p=0.67	83.2±29.3 vs 73.2±17.7, p=0.19	76±59 vs 68±43, p=0.64	1.1±0.4 vs 1.4±0.7, p=0.35	
De Miguel [25] 2023	Retro.	159 (31)	adults	31.5±9.3	Not assessed	Bx						
Bütikofer [24] 2023	Pros.	50 (47)	adults	25.9 (19.5-34.0)	21.8 (16.7-27.8)	Bx						
Jarasvaraparn [26] 2024	Retro.	66	both	24.3±9.3	20.3±7.1	Bx						
Baek [42] 2010	cross-sectional	139	both	19.0±6.3	11.5±4.7	CT						
Shimizu [27] 2016	Retro.	57	both	23.3±9.8	16.2±5.4	CT	24 (19–36) vs 27 (22–32) p=0.59	24 (18–28) vs 28 (19–35) p=0.34		63 (44–87) vs 115 (84–157) p<0.001	1.0 (0.7–1.5) vs 0.9(0.5–1.5) p=0.76	4.6 (4.4–4.8) vs 4.4(4.1–4.8) p=0.18
Song [29] 2018	Retro.	26 (19)	both	13 (10.0-35.0)	10.5 (4-17)	CT	25.6±5.6 vs 24.8±7.3 p=0.831	24.1±7.1 vs 21.6±6.5 p= 0.70		71.5±37.4 vs 73.3 ± 27.1 p=0.66	0.81±0.91 vs 0.78 ± 0.43 p=0.55	4.7±0.3 vs 4.7±0.2 p= 0.890
Wolff [43] 2016	cross-sectional	59	both	19.1 ± 7.5	13.2 ± 7.7	MRI	R=-0.17, p=0.20	R=-0.44, P<0.01	r=0.223, p=0.136	r=-0.441, p=0.01	r=-0.28, p=0.06	r=0.139, p=0.303
Shiina [30] 2020	Pros.	16	adults	31.3 ± 8.5	Not assessed	MRI				r=0.61, p=0.01		r=-0.45, p=0.07
Poterucha [15] 2015	Retro.	50	adults	25 (21-33)	22 (16-26)	MRE				R=0.47, p=0.03		

Alsaied [34] 2019	Retro.	70	adults	24.7 (21.6-32.1)	17.9 (15.1-23.4)	MRE	R = 0.35, p = 0.02	R = 0.37, p = 0.02		R = 0.37, p = 0.03		
Koizumi [35] 2001	Pros.	43	both	17.0 (12.0–25.0)	15.3 (9.7–21.7)	SWE	r= − 0.02 p=0.91	r= 0.23 p=0.13		r= 0.15 p=0.33		r= 0.24 p=0.13
Evans [17] 2017	Retro.	30	both	17 (6–45)	15(1-29)	SWE						
Kim [36] 2018	Retro.	64	both	17.6±5.3	12.1 ± 4.0	SWE	R=0.16, p=0.22	R=0.20, p=0.121		R=0.30, p=0.03	R=-0.21, p=0.09	R=-0.09, p=0.49
Terashi [37] 2019	cross-sectional	79	Ped	10.3±4.9	Not assessed	SWE	R=0.10, p=0.401	R=0.14, p=0.24		R=0.56, p<0.001	R=0.374, p<0.001	
Smaś-Suska [44] 2019	Retro.	54	adults	28.1 ± 19.0	20.4 ± 18.6	SWE	22(16-60) vs 28.5(16-41) p= 0.005	24.5(11-57) vs 28.5(11-51) p=0.08		59(25-170) vs 84(28-255) p=0.01	19.7(9.5–61.6) vs 20.5 (3.5–135) p=0.35	43 (31.5–50) vs 43 (20.5–48.7) p=0.72
An [45] 2020	cross-sectional	66	adults	27.8 ± 6.1	Not assessed	SWE	R=0.50, p <0.01	R=0.40, p = 0.02		R= 0.40 p =0.03		
Nagasawa [28] 2022	cross-sectional	27	both	22.1± 9.4	18.3 ± 8.2	SWE	r=0.26, p=0.20	r=0.14, p=0.47	r=0.07 p=0.74	r=0.14 p=0.49		r=0.34, p=0.081
Gill [46] 2023	Pros.	25	adults	22 (18–29)	18.6±6.2	SWE						
Wu [14] 2014	Retro. & Pros	50 (49)	both	13.1 (2.4–57.7)	9.9 (0.1–32.5)	TE						
Fidai [47] 2017	cross-sectional	27	both	13.5±6.4	8.7±2.5	TE						
Wilson [48] 2018	cross-sectional	152 (133)	both	19.8±9.3	14.1±7.6	TE				r=0.22, p=0.01	r=0.23, p=0.01	
Song [29] 2018	Retro.	26	both	13 (10.0-35.0)	10.5 (4-17)	TE						
Rathgeber [39] 2020	Pros.	76	Ped	11.7 (8.4–14.8)	8.4 (4.6–11.4)	TE	r=-0.45, p<0.01	r=-0.20, p=0.09	r=-0.14, p=0.2	r=0.04, p=0.7	r=0.08, p=0.8	r=0.06, p=0.6
Shin [23] 2022	Retro.	45	adults	25.9 ± 6.5	20.8 ± 4.8	TE	r=0.12 p=0.463	r=0.68, p=0.67	r=0.30 p=0.06	r=0.01, p=0.95	r=0.26 p=0.25	r=0.03 p=0.32
Bütikofer [24] 2023	Pros.	97 (94)	adults	23.1 (18.7-30.6)	18.3 (15.3-25.8)	TE						
Gill [46] 2023	Pros.	25	adults	22 (18–29)	18.6±6.2	TE						

Supplementary Table 4: Results of the association between biochemical parameters and liver fibrosis severity noted by imaging or liver Biopsy. (continue)

Reference	Biochemical parameters†					
	INR	Plt x $10^3/\mu L$	APRI	MELD-XI	Fib-4	Fons index
Wu [16] 2015		171 (127-217) vs 164.5(110-218) p=0.66		9.9(9.4–12) vs 10.2(9.4–13.7) p=0.58		
Munsterman [18] 2019		164(137-186) vs 155(136-191 p=0.96	0.4(0.4-0.5) vs 0.4(0.4-0.6) p=0.53	9.4(9.4-11.0) vs 9.4(9.4-11.1) p=0.87		
Emamaullee [21] 2021	1.6±1.0 vs 1.7±0.7, p=0.04	204±65.8 vs 168±58.4 p=0.01	0.4±0.1 vs 0.5±0.3, p<0.01	10.4 ± 2.1 vs 11.6±3.8, p=0.02	0.4±0.2 vs 0.6±0.4, p<0.01	
Shin [23] 2022	1.1± 0.04 vs 1.3±0.3, p=0.01	207±50 vs 177±50, p=0.11				
De Miguel [25] 2023			0.42 ±0.28 vs 0.63±0.39, p=0.34		1.2 ±1.9 vs 1.5 ±0.8 p=0.12	
Bütikofer [24] 2023		171(112-229) vs 167(135-226) p=0.68		r=0.127, p=0.23		
Jarasvaraparn [26] 2024	1.3 ±0.4 vs 1.4± 0.4 p=0.53	197.65 ±69.02 vs 150.89 ±60.65 p=0.003<br>r=-0.41, p=0.001	0.32 ±0.14 vs 0.64 ±0.78 p=0.02	9.88 ±1.36 vs 11.34 ±4.24 p=0.05	0.71 ±0.42 vs 1.07±0.71, p=0.01<br>r=0.36, p=0.002	
Baek [42] 2010			0.41±0.28 vs 1.19±4.01, =0.003			0.04±3.89 vs 3.68±2.11 p=0.001
Shimizu [27] 2016		17.9(14.0–20.8) vs 14.9(9.3–19.4) p=0.14				6.8(4.4–10.7) vs 11.5(8.0–18.6) p<0.01
Song [29] 2018		220±75 vs 219±46 p= 0.58				
Wolff [43] 2016		r=0.01, p=0.97		r=-0.29, p=0.05	r=-0.32, p=0.02	
Shiina [30] 2020		r=0.001, p=0.99		r=0.51, p=0.04		
Poterucha [15] 2015				R=0.48, p=0.002		
Alsaied [34] 2019		R= −0.3, p = 0.04				
Koizumi [35] 2001		r= − 0.20 p= 0.05	r=0.21, p=0.19		r= 0.25 p=0.11	
Evans [17] 2017			p >0.05	R=0.4, P<0.003		
Kim [36] 2018	r=-0.137, p=0.279	R=-0.02, p=0.99				

Terashi [37] 2019		R=0.39, p<0.001					
Smaś-Suska [44] 2019			0.3(0.2–0.6) vs 0.5(0.2–1.5) p=0.003	9.1 (1–15) vs 10(1.1–19) p=0.13	0.6 (0.3–1.8) vs 0.9(0.3–3.2) p=0.02	3.9 (1.6) vs 4.8 (1.9) p=0.05
An [45] 2020			<0.05		<0.05	
Nagasawa [28] 2022		r=-0.31, p=0.12			r=0.308, p=0.12	
Gill [46] 2023			R=-0.18, p=0.14	R=0.18, p=0.14	R=0.14, p=0.20	
Wu [14] 2014		R=-0.29, p=0.05				
Fidai [47] 2017			r = 0.36, p= 0.01			
Wilson [48] 2018		r=-0.33, p<0.001		r=0.25, p=0.01		
Song [29] 2018		r=-0.47, P=0.02				
Rathgeber [39] 2020		r=-0.39, p<0.01	r=0.26, p=0.04			
Shin [23] 2022	r=0.257, p=0.101	r=-0.05, p=0.77				
Bütikofer [24] 2023		R=-0.28, p=0.01		R=0.13, p=0.23		
Gill [46] 2023			R=0.34, p=0.02	R=0.47, p=0.001		R=0.23, p=0.08

* mean ± SD or median (range or interquartile range).

†Statistics : R, Pearson correlation coeffient; r, Spearman's rank correlation

AST, aspartate transaminase; ALT, alanine transaminase; APRI, aspartate aminotransferase-to-platelet ratio index; Alb, albumin;, ALP, Alkaline phosphatase; Bil, bilirubin, CT, computerized tomography; Ex, examination; GGT, gamma-glutamyl transferase; INR, international normalized ratio; MELD-XI, Model for End-Stage Liver Disease excluding INR; MRE, magnetic resonance elastography; MRI, magnetic resonance imaging; Fib-4, fibrosis 4 index; N, total number of patients; n, number of cases underwent testing; PAWP, pulmonary arterial wedge pressure; Ped, pediatrics; Prosp, prospective; Retro, retrospective; SWE, shear wave elastography; TE, transient elastography

Supplementary Figure 1: Subgroup analysis of Fontan duration for mild and severe liver fibrosis.

(a) Studies on adults

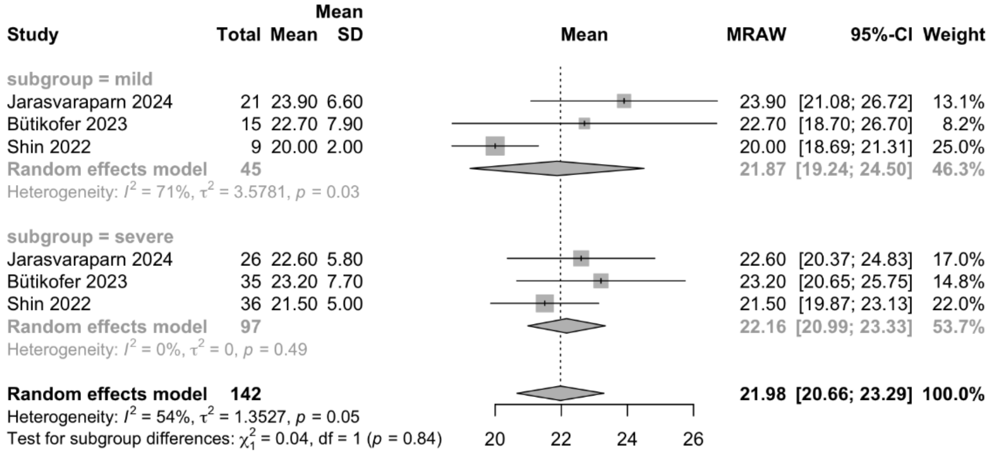

(b) Studies on pediatrics

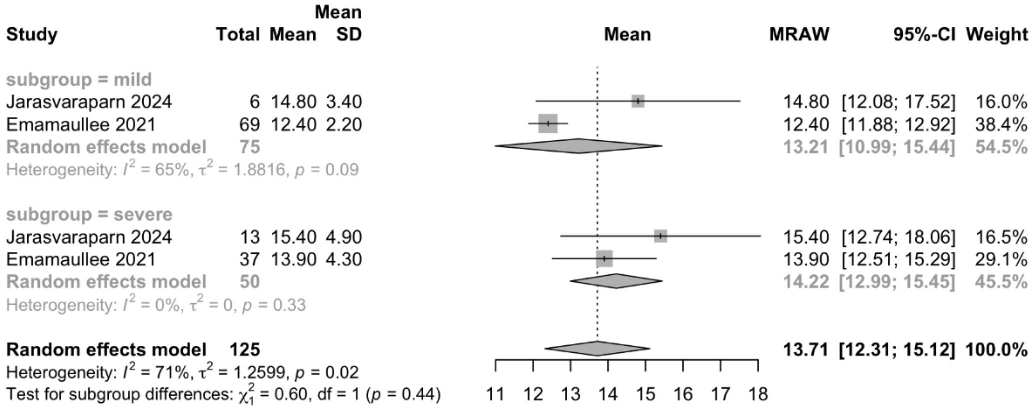

This forest plot shows the mean of Fontan duration (years) across studies. Each study is represented by a square, with size indicating its weight (based on sample size), and horizontal lines showing the 95% confidence intervals (CI). The diamond represents the pooled mean duration with its width indicating the 95% CI. The I^2 statistic measures heterogeneity, and the p-value tests the statistical significance of the pooled estimate.
CI, confidentional interval; MRAW, meta-analysis random-effects Weights; SD, standard deviation

Supplementary Figure 2: Subgroup analysis of age at Fontan operation for mild and severe liver fibrosis

A. Studies with adults

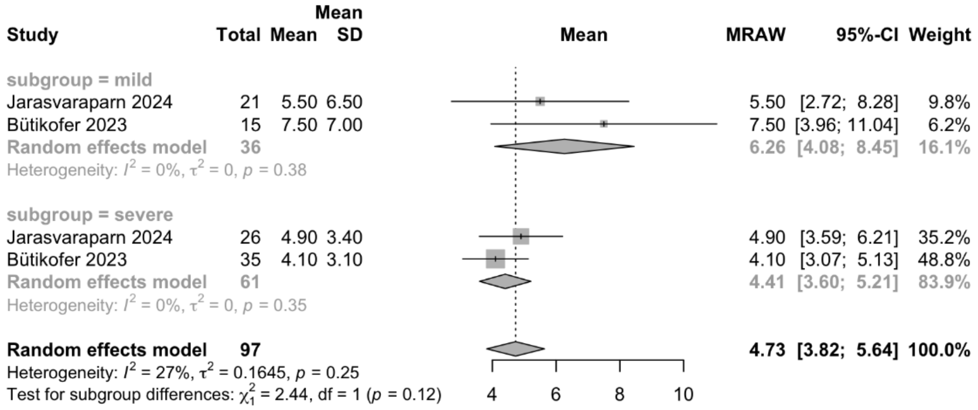

B. Studies with pediatrics

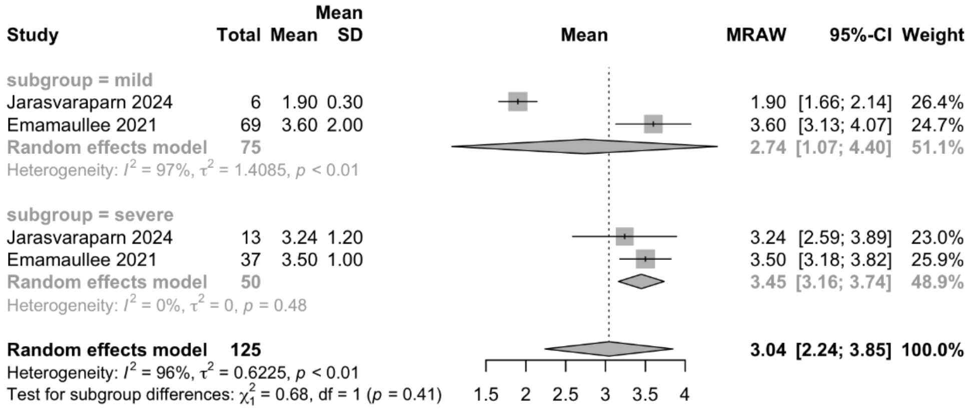

This forest plot shows the mean of age (years) at the Fontan operation across studies. Each study is represented by a square, with size indicating its weight (based on sample size), and horizontal lines showing the 95% confidence intervals (CI). The diamond represents the pooled mean duration with its width indicating the 95% CI. The I² statistic measures heterogeneity, and the p-value tests the statistical significance of the pooled estimate.

CI, confidentional interval; MRAW, meta-analysis random-effects Weights; SD, standard deviation

Supplementary Figure 3: Subgroup analysis of Fontan pressure for mild and severe liver fibrosis.

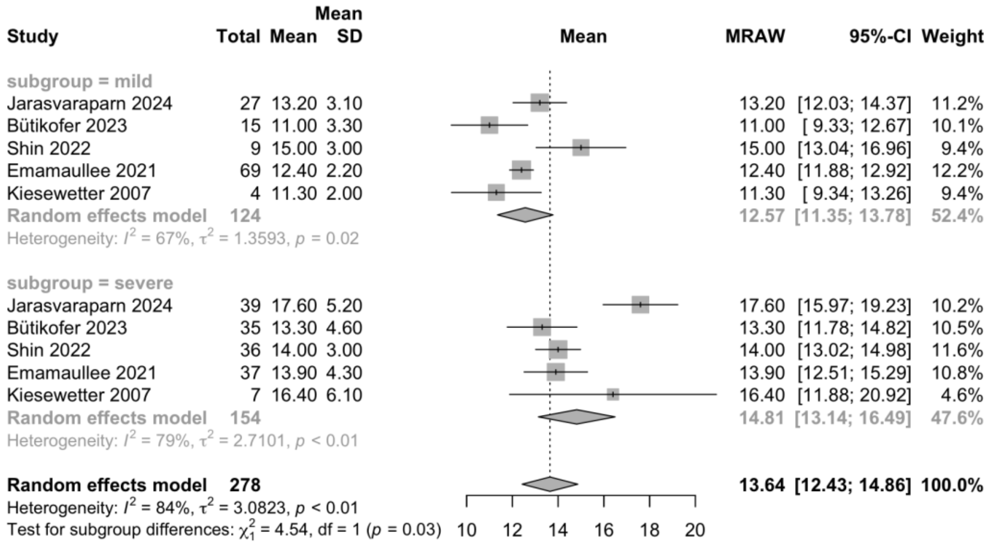

This forest plot shows the mean of Fontan pressure (mmHg) across studies. Each study is represented by a square, with size indicating its weight (based on sample size), and horizontal lines showing the 95% confidence intervals (CI). The diamond represents the pooled mean duration with its width indicating the 95% CI. The I^2 statistic measures heterogeneity, and the p-value tests the statistical significance of the pooled estimate.
CI, confidentional interval; MRAW, meta-analysis random-effects Weights; SD, standard deviation

Supplementary Figure 4: Subgroup analysis of hematologic factors for mild and severe liver fibrosis.

A. Platelet (× 10^3/μL)

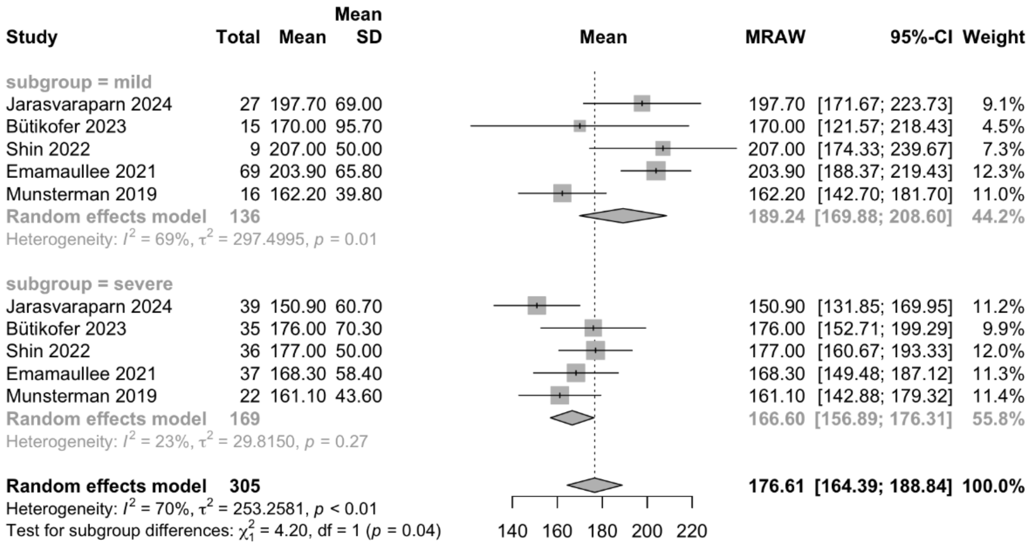

B. GGT (IU/L)

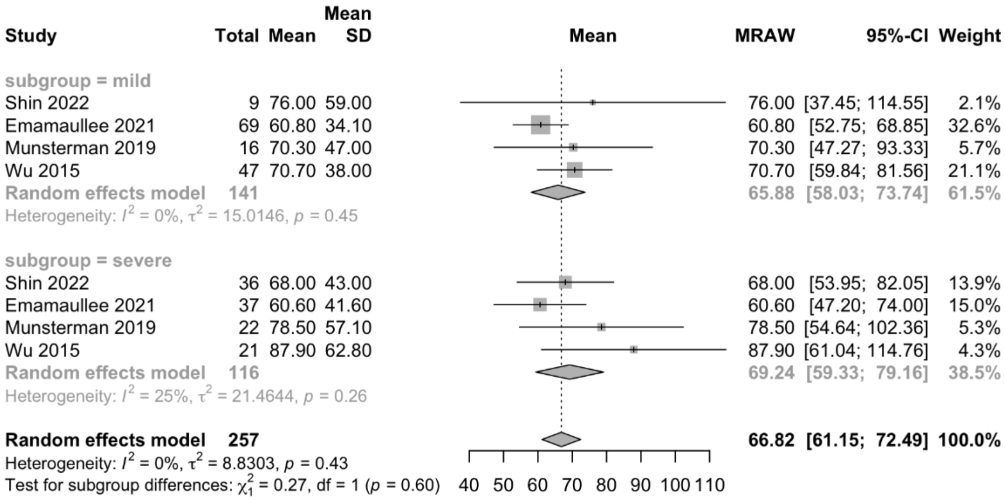

C. Total bilirubin (mmol/L)

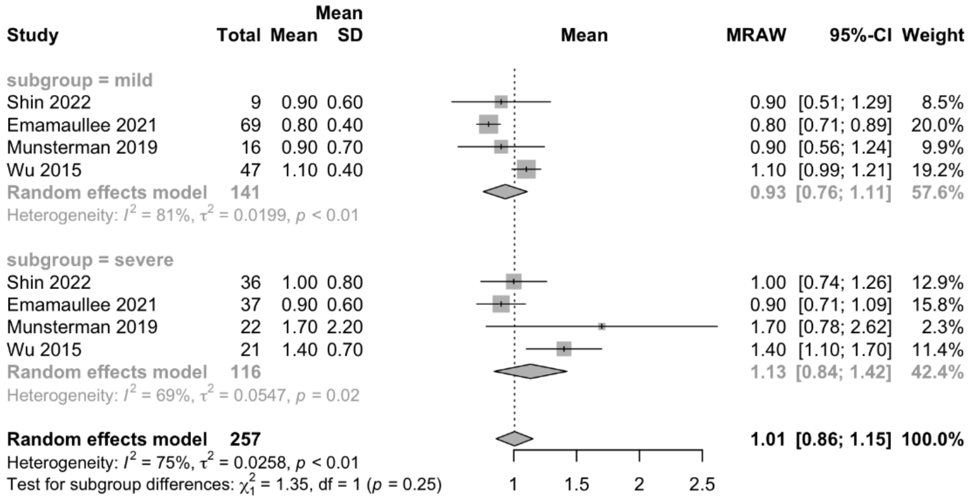

D. APRI

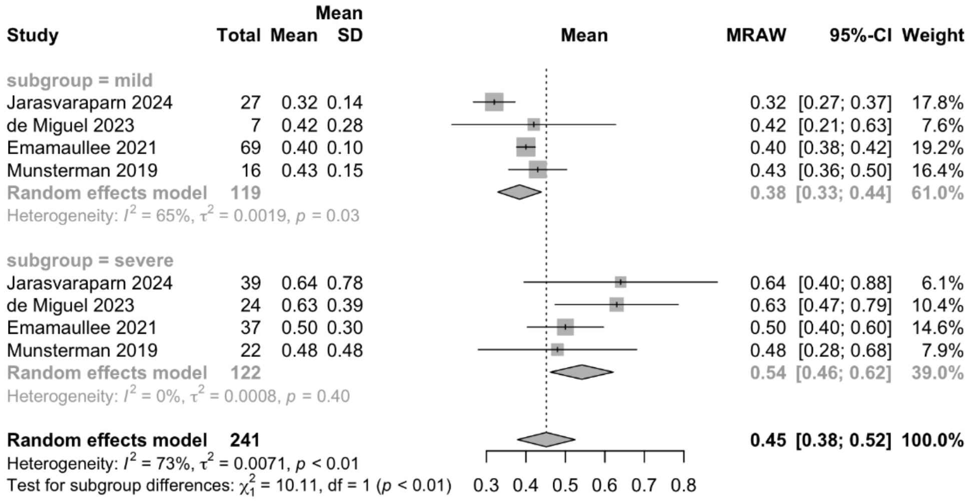

E. Fib-4

(a) Studies on adults

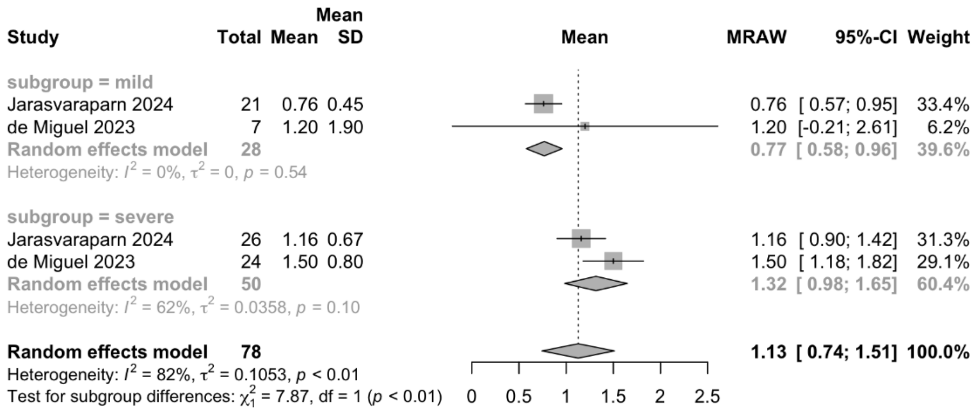

(b) Studies on pediatrics

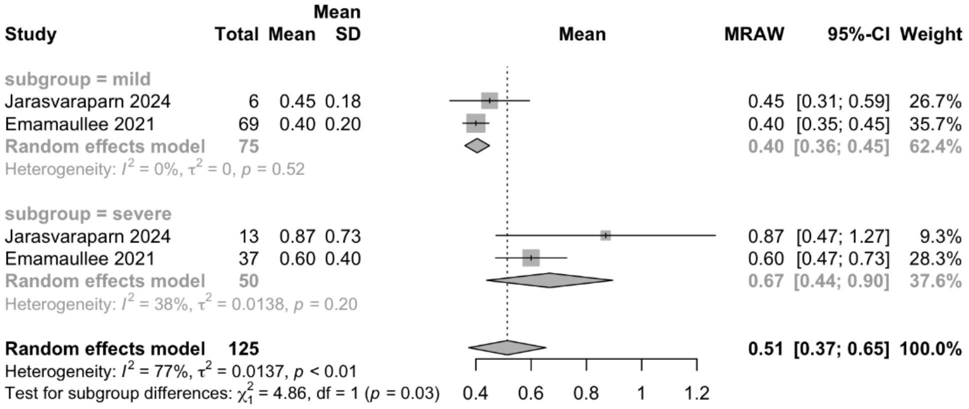

F. MELD-XI

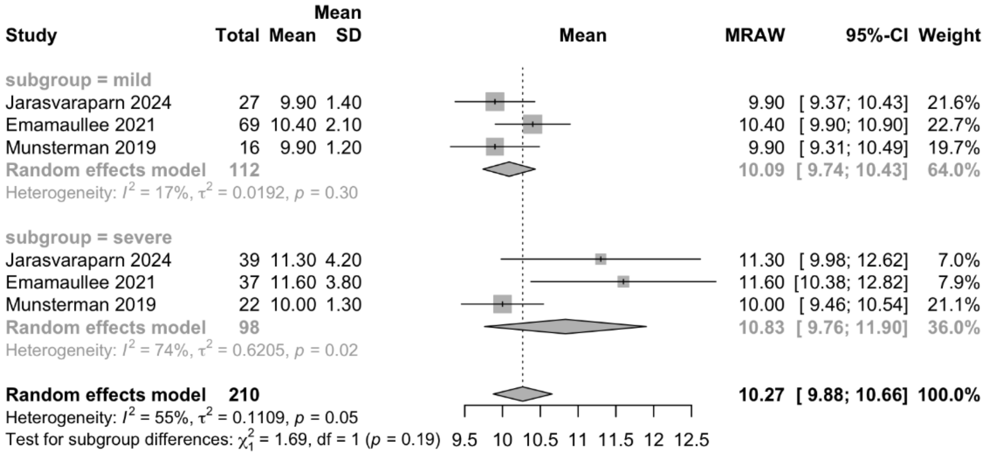

This forest plot shows the mean hematologic factors across studies. Each study is represented by a square, with size indicating its weight (based on sample size), and horizontal lines showing the 95% confidence intervals (CI). The diamond represents the pooled mean duration with its width indicating the 95% CI. The I^2 statistic measures heterogeneity, and the p-value tests the statistical significance of the pooled estimate.

APRI, Aspartate Aminotransferase to Platelet Ratio Index; CI, confidentional interval; Fib-4, Fibrosis-4 Index; GGT, Gamma-Glutamyl Transferase, MELD, Model for End-Stage Liver Disease; MRAW, meta-analysis random-effects Weights; SD, standard deviation
